# Motor Imagery in Asperger Syndrome: Testing Action Simulation by the Hand Laterality Task

**DOI:** 10.1371/journal.pone.0070734

**Published:** 2013-07-23

**Authors:** Massimiliano Conson, Elisabetta Mazzarella, Alessandro Frolli, Dalila Esposito, Nicoletta Marino, Luigi Trojano, Angelo Massagli, Giovanna Gison, Nellantonio Aprea, Dario Grossi

**Affiliations:** 1 Neuropsychology Laboratory, Department of Psychology, Second University of Naples, Caserta, Italy; 2 Department of Neuromotor Physiology, Scientific Institute Foundation Santa Lucia, Rome, Italy; 3 Service of Neuropsychiatry, Second University of Naples, Naples, Italy; 4 Scientific Institute I.R.C.C.S. “Eugenio Medea” Regional Branch of Ostuni, Brindisi Department of Neurorehabilitation 2, Child Psychiatry, Brindisi, Italy; 5 Centro Medico Riabilitativo Pompei s.r.l., Pompei, Italy; University of California, Merced, United States of America

## Abstract

Asperger syndrome (AS) is a neurodevelopmental condition within the Autism Spectrum Disorders (ASD) characterized by specific difficulties in social interaction, communication and behavioural control. In recent years, it has been suggested that ASD is related to a dysfunction of action simulation processes, but studies employing imitation or action observation tasks provided mixed results. Here, we addressed action simulation processes in adolescents with AS by means of a motor imagery task, the classical hand laterality task (to decide whether a rotated hand image is left or right); mental rotation of letters was also evaluated. As a specific marker of action simulation in hand rotation, we assessed the so-called biomechanical effect, that is the advantage for judging hand pictures showing physically comfortable versus physically awkward positions. We found the biomechanical effect in typically-developing participants but not in participants with AS. Overall performance on both hand laterality and letter rotation tasks, instead, did not differ in the two groups. These findings demonstrated a specific alteration of motor imagery skills in AS. We suggest that impaired mental simulation and imitation of goal-less movements in ASD could be related to shared cognitive mechanisms.

## Introduction

Autism Spectrum Disorders (ASD), including autism and Asperger syndrome (AS), are characterized by developmental impairments in communication and social interaction, together with repetitive stereotyped behaviours [1]. Typically, individuals with AS display pervasive difficulties in behavioural control and in social understanding and communication, and show interpersonal awkwardness; on formal tests they tend to exhibit relative strengths in verbal skills and rote learning, but weaker visuomotor and conceptual learning abilities [2], [3].

Over the last decade, several studies suggested that ASD is linked to an alteration of action simulation processes [4]–[6]. Different views of simulation are available in literature, but almost all share the idea that it involves sensorimotor representations, that are also activated through observation or imitation of others' behaviour [7], or by imagining one's own or others' actions (for a review see [8]).

Action simulation has been extensively investigated in ASD by means of imitation tasks, but with contrasting results [9]. Some studies reported defective performance and altered patterns of sensorimotor brain activity during imitation [10], [11], whereas other reported spared imitative abilities [12], and typical patterns of sensorimotor cortical activation during observation and execution of hand actions [13], [14]. Recent behavioural studies shed new light in this issue by demonstrating a dissociation between goal-directed and goal-less imitation in ASD: individuals with ASD show relatively spared performance in imitating goal-directed movements and actions with objects, whereas they show impaired imitation of goal-less or meaningless actions [9], [12], [15]–[18].

As recalled above, action simulation implies that the same motor representations are involved not only in imitation or action observation but also in motor imagery [7], [8]. It is widely held that motor imagery recruits neural resources typically employed during one's own body movements [19]–[24]. On a behavioural ground, it has been demonstrated that the time required to mentally simulate a movement mimics that needed to perform the corresponding motor act [25].

**Figure 1:**
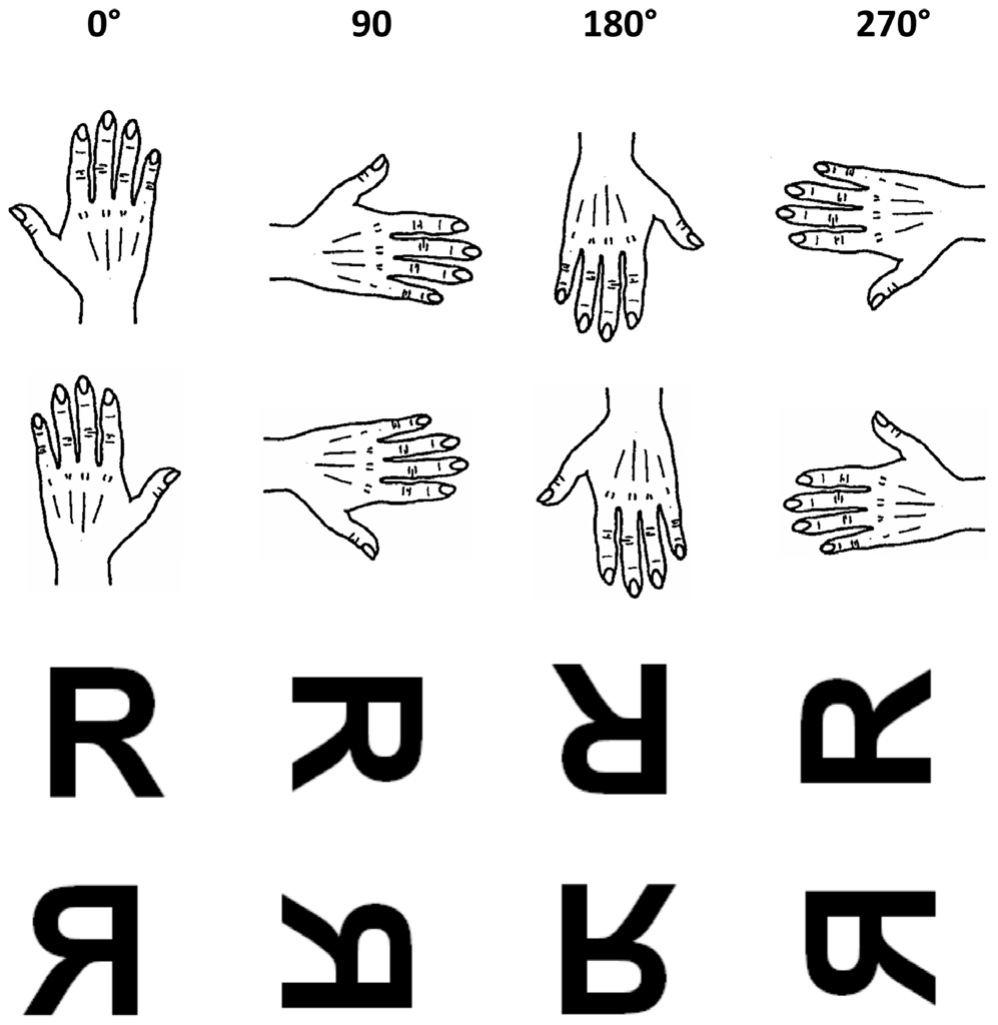
Stimuli used in the two mental rotation tasks: hand laterality task (first row right hand; second row left hand) and letter rotation (third row letter “R” in canonical orientation; fourth row letter “R” in mirror-reversed orientation).

In the present study, we aimed at investigating action simulation in ASD through a motor imagery task. Motor imagery is classically assessed by means of the hand laterality task in which participants have to decide whether a visual stimulus presented in different angular orientations portrays a left or a right hand (see Figure 1) [26]–[29]. Psychophysical studies on healthy participants demonstrated that subjects perform the task by mentally simulating movements of their own hands [27]–[28]. Actually, performance on the hand laterality task is strongly influenced by anatomic/biomechanical constraints, with faster response times when subjects have to judge hand pictures showing physically comfortable versus physically awkward positions [27]–[28]. More precisely, participants are faster in judging a 90° oriented left hand (fingers pointing to the right; medial orientation with respect to the body sagittal plane) than a 90° oriented right hand (lateral orientation with respect to the body sagittal plane); analogously, participants show an advantage when judging a 270° oriented right hand (fingers pointing to the left; medial orientation) than a 270° oriented left hand (lateral orientation). The significant effect of biomechanical constraints on motor imagery is thought to be a hallmark of the embodied nature of simulated movements [19], [21], [27]–[31], and is not found in brain-damaged patients with severe lesions of the motor system [19], [20].

Here, we assessed action simulation in individuals with AS by the hand laterality task. If action simulation is defective in AS, then we should find no evidence of the biomechanical effect on the participants' performance; such a finding would imply that AS individuals resort to visuospatial processes to solve the task, as in mental rotation of non-corporeal stimuli. In recent years, several studies provided convergent evidence that visuospatial mental transformation is spared or even enhanced in ASD compared to typically-developing individuals [32]–[36], when assessed on concrete objects, three-dimensional figures, letters or geometrical shapes [37], [38]. On this basis, we also tested AS individuals on mental rotation of letters; by these means, we could ascertain whether a defect of action simulation could be independent from the ability to mentally rotate non-corporeal stimuli.

## Methods

### Ethics Statement

All of the participants to this study completed the experimental tasks that had been previously approved by the local ethical committee (Comitato Etico del Dipartimento di Psicologia della Seconda Università di Napoli) and were conducted according to the Helsinki declaration. Written informed consent was obtained from the parents of each participant involved in our study.

### Participants

Forty-eight right-handed adolescents were recruited for the study; twenty-four individuals with AS (21 males and 3 females) and twenty-four controls (20 males and 4 females). In all participants diagnosis of AS was reached after a multidisciplinary assessment by a neuropsychiatrist and a clinical psychologist trained in evaluation of individuals with neurobehavioural disorders according to DSM-IV-TR criteria. Clinical diagnosis was validated by means of the Autism Diagnostic Interview-Revised (ADI-R) [39] and the Autism Diagnostic Observation Schedule (ADOS) Module 3 [40]; general intelligence was measured by means of the Wechsler Intelligence Scale for Children-Revised (WISC-R) [41]. Individuals with a history of epilepsy, neurological abnormalities, genetic syndromes, general learning disability, known history of significant head injury, or psychosis were excluded from the study. Typically-developing adolescents, without history of neurological or psychiatric diagnosis and matched for age and gender with the AS group, were recruited from secondary schools in Naples. Cognitive level of the control group was assessed on Raven's Progressive Matrices [42], [43]; estimated IQ did not differ from the mean IQ of the AS group (t = .396; p = .694) (see Table 1).

**Table 1:** Demographics of the participants. All data are given as mean ± standard deviation (range).

	N	Chronological age	Verbal IQ	Non-verbal IQ	Total IQ
AS group	24	13.4±1.3 (12–16)	100.5±4.6 (95–109)	99.5±6.1 (91–116)	100±5.1 (91–106)
Typical group	24	13.3±1.4 (12–16)	-	-	99.4±4.4 (90–105)

### Experimental tasks

The experiment consisted of two tasks: hand laterality judgement and letter rotation task. In the hand laterality judgement, stimuli were left or right hands portrayed from back at 0° (upright), 90° (clockwise), 180° (upside down) or 270° (90° anticlockwise) orientations; in the letter rotation task, one capital letter (“R”) was presented in canonical or mirror-reversed form, in the same four orientations (0°, 90°, 180° or 270°) as in the hand rotation (see Figure 1).

In both tasks, stimuli were large approximately 6 cm along the widest axis (about 5.5° of visual angle at a viewing distance of 60 cm) and were presented at the centre of a 15″ computer screen. Each trial began with the presentation of a fixation point for 500 ms and, after a delay (300 or 500 ms), the stimulus was presented and remained on view until response completion.

In the hand laterality task, subjects were required to decide whether the visual stimulus corresponded either to a left or a right hand. In the letter rotation task participants had to decide whether the letter was shown in its canonical or mirror-reversed form.

Each task consisted of 48 randomised trials: in the hand rotation, 6 trials were presented for each combination of hand laterality (left or right) and orientation (0°, 90°, 180° or 270°); in the letter rotation, 6 trials were presented for each combination of type of letter (canonical or mirror-reversed) and orientation.

Participants gave their responses by pressing one of two centrally located keys with their index and middle fingers of the right hand; the stimulus-response association for each task was counterbalanced across participants. The left hand was placed palm down next to the keyboard, and both hands were covered with a black cloth. Participants were encouraged to respond as fast and correctly as possible; we recorded both Reaction Times (RTs, in milliseconds, ms) and error rates. Stimulus presentation and data collection were controlled by a PC using Cedrus SuperLab v.4.

The order of the two tasks was counterbalanced across participants. Each task was divided in two blocks; a 3-min pause was allowed between the two blocks, and after each task. A training period preceded the experiment. Before starting each task, at least eight practice trials were given; during practice, if a wrong response was provided, a feedback appeared on the monitor screen and the trial was repeated. Experimental session started only if the participants provided at least six consecutive correct responses.

### Statistical analysis

Mean RTs and error rates were calculated (data are freely available upon request; please contact the corresponding author) and then submitted to Analysis of Variance (ANOVA). First, we specifically searched for the biomechanical effect in the hand laterality task by means of a three-way mixed-design ANOVA with hand laterality (left or right) and stimulus orientation (90° or 270°, the two spatial orientations that, together with hand laterality, can best reveal the biomechanical effect) as within-subject factors, and group (AS or controls) as a between-subject factor. Then, to compare general mental rotation abilities in the two groups, we conducted a three-way mixed-design ANOVA with task (hands or letters) and stimulus orientation (0°, 90°, 180° or 270°) as within-subject factors, and group (AS or controls) as a between-subject factor. These two ANOVAs were performed separately on mean RTs (for correct responses) and error rates. Finally, following previous studies [37], to test whether the participants used a mental rotation strategy, we performed planned linear contrasts on participants' correct RTs for stimulus orientations from 0° to 180° on each experimental task.

## Results

### Effect of biomechanical constraints on hand rotation

The three-way mixed ANOVA on RTs assessing the biomechanical effect showed a significant main effect of hand laterality, F(1,46) = 7.584, p = .008, η^2^_p_ = .142, with faster RTs when judging right (mean  = 2441 ms, SEM = 75.5) than left hand (mean  = 2659 ms, SEM = 56.4). Moreover, results showed significant first-order interactions between hand laterality and group, F(1,46) = 21.947, p = .0001, η^2^_p_ = .323, and between hand laterality and stimulus orientation, F(1,46) = 11.829, p = .001, η^2^_p_ = .205; the second-order interaction among hand laterality, stimulus orientation and group was also significant, F(1,46) = 8.171, p = .006, η^2^_p_ = .151. No other main effect or interaction was significant (all p>.05).

Post-hoc comparisons (paired t-tests) on the interaction between hand laterality and group showed that individuals with AS were faster in judging right (mean  = 2501 ms, SEM = 79.1) than left hand stimuli (mean  = 2736 ms, SEM = 82.4; t = −2.575, p = .017), whereas judgments on right (mean  = 2548 ms, SEM = 107.4) and left hands (mean  = 2480 ms, SEM = 94.8;) did not differ in controls (t = .928, p = .363).

**Figure 2:**
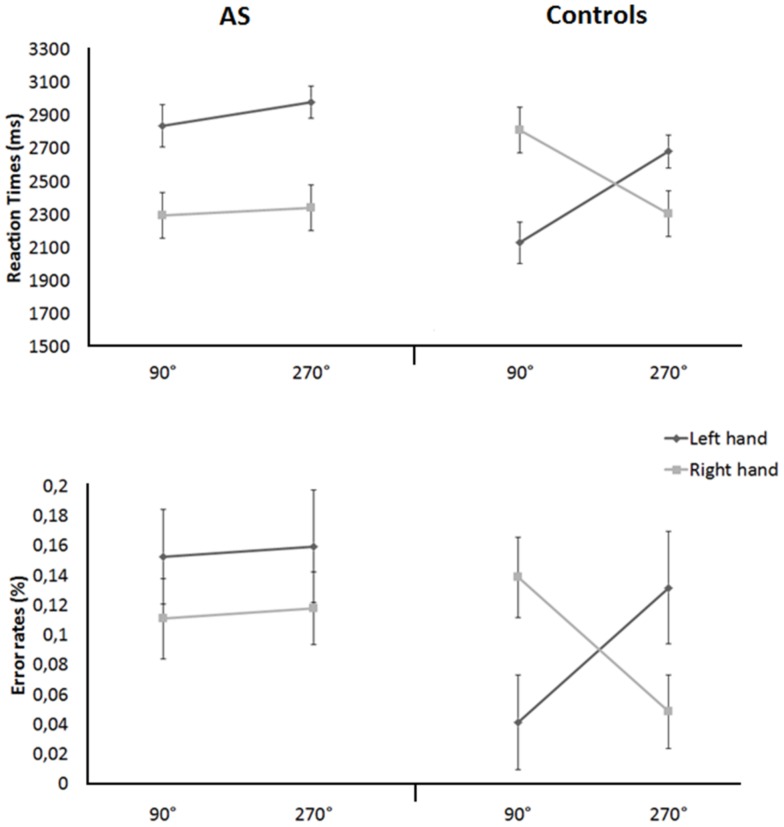
Mean RTs on the hand rotation task for the two groups by degree of stimulus orientation and hand laterality (i.e., biomechanical effect); bars represent SEM.

Post-hoc comparisons (paired t-tests) on the interaction between hand laterality and stimulus orientation showed that participants were faster in judging right (mean  = 2324 ms, SEM = 95.6) than left 270° oriented hands (mean  = 2832 ms, SEM = 72.3; t = −4.538, p = .0001), whereas no difference was found between left (mean  = 2485 ms, SEM = 102.1) and right 90° oriented hands (mean  = 2557 ms, SEM = 103.6; t = .482, p = .632). Relevantly, post-hoc comparisons (paired t-tests) on the interaction among stimulus laterality, stimulus orientation, and group (Figure 2, upper row) showed that typically developing participants were significantly faster in judging left than right 90° oriented hands (t = 3.408, p = .002), whereas the opposite was true at 270° orientation (t = −2.824, p = .010). This finding is the behavioural mark of the biomechanical effect. Adolescents with AS did not show the same pattern, but were significantly faster in responding to right than left hand stimuli at both 90° (t = −4.138, p = .0001) and 270° orientations (t = −3.577, p = .002).

The same three-way mixed ANOVA design as above applied on error rates showed a significant first-order interaction between hand laterality and stimulus orientation, F(1,46) = 4.504, p = .039, η^2^_p_ = .089, and a significant second-order interaction among hand laterality, stimulus orientation and group, F(1,46) = 4.605, p = .036, η^2^_p_ = .090. No other main effect or interaction was significant (all p>.05).

Post-hoc comparisons (paired t-tests) on the interaction between hand laterality and stimulus orientation did not show significant differences between judgments on left and right 90° oriented hands (left: mean  = .10, SEM = .02; right, mean  = .13 ms, SEM = .01; t = .955, p = .344), whereas error rates were significantly lower with right than left 270° orientated hands (left: mean  = .15, SEM = .03; right: mean  = .08, SEM = .02; t = −2.172, p = .035). More relevant here, post-hoc comparisons (paired t-tests) on the interaction among stimulus laterality, stimulus orientation, and group (Figure 2, lower row) showed that typical adolescents were significantly more accurate in judging left than right 90° oriented hands (t = 3.077, p = .005), whereas the opposite was true at 270° orientation (t = −2.505, p = .020), consistent with the biomechanical effect. Instead, AS' error rates on right than left hand were not affected by stimuli's orientation (p>.05).

### Effect of stimulus orientation and task on mental rotation performance

**Figure 3:**
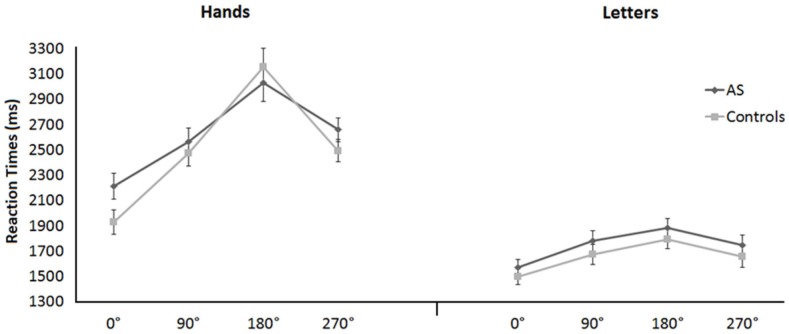
Mean RTs of mental rotation of hands and letters plotted against the four stimulus orientations separately in the two groups; bars represent SEM.

Overall performance of individuals with AS and controls on the two experimental tasks is reported in Table 2. The three-way mixed-design ANOVA on RTs showed significant main effects of task, F(1,46) = 232.035, p = .0001, η^2^_p_ = .835, with slower responses on the hand laterality judgment (mean  = 2566 ms, SEM = 57.8) than on letter rotation (mean  = 1701 ms, SEM = 21.5), and of stimulus orientation, F(3,138) = 56.649, p = .0001, η^2^_p_ = .552, with faster responses to 0° (mean  = 1805 ms, SEM = 41.2) than to the other orientations (90°: mean  = 2125 ms, SEM = 42.6; 180°: mean  = 2466 ms, SEM = 54.9; 270°: mean  = 2137 ms, SEM = 41.7). Moreover, we found a significant interaction between task and stimulus orientation, F(3,138) = 19.236, p = .0001, η^2^_p_ = .295 (see planned linear contrasts and Figure 3). No other main effect or interaction was significant (all p>.05); notably, the factor group did show any interaction with the other considered variables (task or stimulus orientation).

**Table 2:** Mean RTs (and standard deviations in brackets) of participants with AS and typical controls separated for right and left hands across the four orientations.

	AS				Controls			
	0°	90°	180°	270°	0°	90°	180°	270°
Hands								
*Right*	2174.7	2298.8	3190.5	2342.7	1885.1	2815.4	3189.1	2306.3
	(541.8)	(578.9)	(715.4)	(720.4)	(652.2)	(760.8)	(745.1)	(614.6)
*Left*	2254	2839.5	2869.4	2981.4	1978.2	2131.5	3129.6	2684
	(693)	(553.1)	(994.4)	(424.1)	(617.3)	(673.2)	(822.1)	(536.4)
Letters								
*Canonical*	1438	1704.6	1875.8	1683.6	1386.2	1624.2	1794.2	1640.9
	(283.3)	(389.6)	(226.4)	(386.8)	(286.1)	(304.5)	(401.7)	(424.9)
*Reversed*	1711.6	1861.9	1889.9	1787.1	1615.9	1725	1797.3	1669.5
	(346.4)	(460.5)	(334.5)	(384.4)	(297.2)	(409.8)	(334.6)	(380.8)

The same three-way mixed-design ANOVA design as above performed on error rates (see Table 3) showed a significant main effect of stimulus orientation, F(3,138) = 12.581, p = .0001, η^2^_p_ = .215, with faster responses to 0° (mean  = .05, SEM = .01) than to the other orientations (90°: mean  = .11, SEM = .02; 180°: mean  = .16, SEM = .02; 270°: mean  = .10, SEM = .01). No other main effect or interaction was significant (all p>.05), but it is worth noting that consistent with RTs data participants made more errors on the hand laterality task (mean  = .12 ms, SEM = .02) than on letter rotation (mean  = .09 ms, SEM = .01); moreover, the factor group did not show any interaction with task or stimulus orientation.

**Table 3:** Mean error rates (and standard deviations in brackets) of participants with AS and typical controls separated for right and left hands across the four orientations.

	AS				Controls			
	0°	90°	180°	270°	0°	90°	180°	270°
Hands								
*Right*	.06	.11	.17	.12	.04	.14	.17	.05
	(.12)	(.14)	(.22)	(.14)	(.09)	(.12)	(.26)	(.09)
*Left*	.09	.15	.18	.16	.05	.04	.18	.13
	(.16)	(.19)	(.25)	(.21)	(.08)	(.11)	(.21)	(.16)
Letters								
*Canonical*	.04	.09	.15	.10	.03	.07	.14	.09
	(.10)	(.16)	(.12)	(.12)	(.07)	(.21)	(.16)	(.16)
*Reversed*	.06	.12	.16	.13	.05	.10	.15	.11
	(.12)	(.17)	(.23)	(.17)	(.09)	(.16)	(.20)	(.19)

Planned linear contrasts performed on participants' correct RTs for stimulus orientations from 0° to 180° showed a significant linear increase in RTs as stimulus orientation increased when both individuals with AS and controls mentally rotated hands (AS: F(1,23) = 24.317, p = .0001, η^2^_p_ = .514; controls: F(1,23) = 149.289, p = .0001, η^2^_p_ = .867) and letters (AS: F(1,23) = 19.909, p = .0001, η^2^_p_ = .464; controls: F(1,23) = 15.040, p = .001, η^2^_p_ = .395). Figure 3 shows RTs of the two groups on hand and letter rotation plotted against the four stimulus orientations (0°, 90°, 180°, and 270°).

## Discussion

In the present study, we used the hand laterality task to assess action simulation in AS. Results showed a significant biomechanical effect in typically-developing controls but not in individuals with AS. This finding would suggest that AS adolescents do not implement motor strategies to mentally simulate hand movements [44]. However, overall RTs and error rates did not differ between AS and typical participants on both hand laterality task and mental rotation of letters; the linear trend in RTs related to increasing stimulus orientation suggested that both groups resorted to visuospatial rotation in mental transformation of hands and letters [37]. In other words, we do not argue that individuals with AS cannot perform motor imagery, but rather we suggest that they do not activate action simulation processes to mentally transform body parts.

Until now, almost all studies on mental transformation processes in ASD required participants to mentally rotate concrete or abstract objects. Silk et al. [35] observed no difference between autistics and controls in mental rotation of three-dimensional figures, although functional magnetic resonance findings revealed a dysfunction of frontal structures in autistic individuals during the task. Analogously, Beacher et al. [36] found that men and women with AS performed mental rotation and verbal fluency tasks as well as typically-developing controls, but neuroimaging data showed in AS males stronger activations of posterior and frontal regions during mental rotation than controls. Further behavioural studies confirmed the finding of efficient visuospatial mental rotation abilities in ASD. For instance, Falter et al. [34] demonstrated that individuals with ASD performed even better than typical controls on mental rotation of three-dimensional figures. Analogously, Hamilton et al. [33] found that children with ASD performed better than typical children on mental rotation of objects (but performed worse on a visual perspective taking task). More recently, Soulières et al. [32] observed that individuals with ASD could be more accurate than typically-developing controls in mentally rotating different kinds of stimuli (geometrical shapes, letters and hands). However, the authors did not search for the biomechanical effect in hand rotation, and thus could not directly tackle the issue of action simulation.

The present findings concur with evidence reviewed above in demonstrating that individuals with ASD have spared (or even enhanced) mental transformation skills if one considers overall behavioural performance. On the contrary, we argue that peculiar alterations of mental rotation abilities, and in particular of action simulation processes, are present in subjects with ASD.

In a recent meta-analysis of neurofunctional studies on motor imagery, Hétu et al. [24] suggested that laterality judgments mainly rely on activation of multisensory (visual and proprioceptive) representations of one's own effector. A large corpus of neuropsychological evidence in brain-damaged patients demonstrated that an alteration of an integrated body representation can be related to both motor imagery and imitation defects [45]–[48]. For instance, Buxbaum et al. [46] described a patient with progressive apraxia who showed a prominent impairment in imitation of meaningless movements, and also showed a defective performance on the hand laterality task. Analogously, Schwoebel et al. [48] demonstrated in a group of left hemisphere stroke patients that performance on imitation of meaningless gestures selectively correlated with performance on the hand laterality task. It is worth noting here, that the hand laterality task implies mental simulation of hand movements not related to an object or a goal; in other words, it requires mental imagery of meaningless, intransitive and goal-less movements. It has also been shown that brain-damaged apraxic patients failing on imitation of object-related gestures were impaired on a modified hand laterality task requiring mental simulation of object-related, transitive actions [47]. Taken together, these findings suggest that impairments of mental simulation and imitation of meaningless, intransitive actions can be ascribed to shared defective cognitive and neural mechanisms [24], [48].

Dual-route models of imitation posit that meaningless gestures are reproduced via a direct route directly mapping visual inputs onto motor outputs [9], [49], [50]. A dysfunction of such imitation route in individuals with ASD has been claimed to account for their impaired goal-less imitation [9]. The present findings strongly support and extend these observations, by revealing that not only imitation but also mental imagery of goal-less movements is impaired in ASD. It remains to be explored whether disorders of action simulation in ASD also extends to motor imagery of goal-directed and transitive movements, that have been extensively investigated in the imitation domain (see Caspers et al. [51] for a meta-analysis).

Our evidence of impaired simulation of one's own actions in ASD might parallel ASD individuals' defective performance in visual perspective taking (VPT) tasks [33]. In particular, ASD individuals have difficulties in understanding how another person perceives a given object from his/her viewpoint (second level of VPT, VPT-2) [33]. Recently, Kessler and Wang [52] reported that individuals with low autistic traits, the so-called “empathisers” as identified by means of the Autistic Quotient [53], were more prone to mentally rotate their own body to align with another's body; individuals with high autistic traits (“systemisers”), instead, adopted alternative strategies, likely object rotation, to solve the task. Although mental simulation of one's own movements in VPT and motor imagery tasks are both related to sensorimotor processes, and although our finding fit with those on VPT-2 [33], [52], available data do not allow to clearly establish whether or not they share the same cognitive and neurofunctional bases [54], [55]. Further investigation is necessary to directly test commonalities and differences between embodied simulation processes involved in motor imagery and visual perspective transformations.
